# Synthesis of new benzothiazole derivatives with in-depth *In-vitro, In-vivo* anti-oxidant, anti-inflammatory and anti-ulcer activities

**DOI:** 10.1371/journal.pone.0337639

**Published:** 2026-01-30

**Authors:** Ayesha Khan, Neelum Gul Qazi, Arooj Mohsin Alvi, Safia Obaidur Rab, Kashif Iqbal, Humaira Nadeem

**Affiliations:** 1 Department of Pharmaceutical Chemistry, Riphah Institute of Pharmaceutical Sciences, Riphah International University, Islamabad, Pakistan; 2 Department of Pharmacy, Iqra University, Islamabad, Pakistan; 3 Faculty of Pharmacy, Ibadat International University, Islamabad, Pakistan; 4 Department of Clinical Laboratory Sciences, College of Applied Medical Science, King Khalid University, Abha, Saudi Arabia; University of Westminster - Regent Street Campus: University of Westminster, UNITED KINGDOM OF GREAT BRITAIN AND NORTHERN IRELAND

## Abstract

Benzothiazoles (BTZ) possess various medicinal benefits and in this study, we have synthesized benzothiazole derivatives by substituting its side chains, and analyzed its spectral analysis along with its antioxidant and anti-inflammatory activity. In-vitro antioxidant activity of the benzothiazole derivatives indicated strong antioxidant potential of **3b**, **3d** and **3e** which were then evaluated for their anti-inflammatory potential in mice’s paw edema and ethanol-induced gastric ulcer (GU). The compounds demonstrated strong anti-inflammatory ability in paw edema as well as ethanol-induced gastric ulcer. This was further confirmed by H&E staining and suppressed inflammatory mediators as tumor necrotic factor (TNF-α), nuclear factor B (p-NFkB), and cyclooxygenase-2 (COX-2). The antioxidant ability was assessed by measuring catalase (CAT), glutathione-S-transferase (GST), glutathione (GSH), superoxide dismutase (SOD), and lipid peroxidation (LPO) levels in both edema and ulcer models. Additionally, gastric lesions were detected in the ulcer and low Ulcer index confirmed anti-ulcer ability. These results suggest new benzothiazoles which can be further analyzed through clinical studies.

## Introduction

Benzothiazoles possess characteristic structural features and a wide range of biological activities, highlighting benzothiazole as an important pharmacophore in drug discovery [1]. It can be widely obtained from both marine and terrestrial natural compounds and demonstrates a strong biological profile, attracting researchers’ attention to design new bioactive derivatives [2]. Hence, it has gained a lot of importance in the field of medicinal chemistry [3]. It exhibits many useful therapeutic activities such as antitubercular, antimicrobial, antimalarial, anticonvulsant, anthelmintic, analgesic, anti-inflammatory, antidiabetic, and antitumor activity, etc [4]. Considering their pharmacological benefits from previous data, we synthesized various benzothiazole derivatives by substituting their side chains and analyzed their antioxidant profile, along with their anti-inflammatory activity, which may impart it gastroprotective ability in gastric ulcers.

Gastric ulcer (GU) is characterized by a deep lesion affecting mucosa and the muscularis mucosa of the gastric walls. The estimated prevalence of gastric ulcer disease in the general population is 5–10% [5]. Extensive studies have proved that gastric mucosa of the stomach is itself capable of self-protection by various innate mechanisms such as alkaline secretion of the mucosa, epithelial blood flow, mucous secretion, etc. All these mechanisms combined provide ample protection to the gastric mucosa against every injury and mucosal insults [6]. The pathological condition arises from an imbalance between the protective agents (e.g., the secretion of mucus, mucosal barrier, and regeneration of cells), and aggressors present in the gastric mucosa (e.g., acid–pepsin secretion) and is linked to the digestive process as well as some other factors like consumption of excess alcohol, chronic use of NSAIDs, stress and *H. pylori* infections. Currently, it has been shown that aging is also associated with a high incidence of gastric ulcers in the elderly along with excess use of NSAIDs, alcohol intake, and stress [7]. The exact mechanism is not clear but it is firmly believed that the acute phase of gastric ulcer presents an imbalance between inflammatory response and oxidation, which has been proved by the up-regulation of pro-inflammatory cytokines like TNF-α, IL-1β, and IL-6, recruitment of mononuclear cells and neutrophils as well as free radicals generation [6].

Free radical generation is a hallmark of many diseases, including gastric ulcers. Moreover, many researchers have established a direct connection between free radical generation and inflammatory cascade activation [8], which forms the basis of many therapeutic strategies for the treatment of gastric ulcers. Gastric ulcers are often accompanied by necrosis, reduced blood flow, release of inflammatory mediators, neutrophil infiltration, and oxidative stress. Inflammation is a natural defense mechanism of the body recruited to protect the body from invasive stimuli [9]. The common manifestation of inflammation includes edema formation, leukocyte infiltration, and granuloma development [7]. If unchecked, excessive inflammation may lead to an exacerbated response as indicated by the generation of cytokines such as NFkB, and recruitment of proinflammatory mediators such as TNF-α, IL-1β, and COX-2 [10]. These, in turn, can initiate a variety of reactions that cause further aggravation of the diseased state of the mucosa.

Due to the high prevalence and low quality of life after ulcer formation, research is still underway to find a cure that provides maximum gastric protection and minimal side effects. Many agents inhibiting activation of H^+^/K^+^-ATPase, proton pumps, and H2-receptors have been marketed, concurrently given with agents that reduce stress, overall inhibiting excessive acid secretion and protecting gastric mucosa [11]. However, extended usage has been linked to several side effects including, but not limited to, arrhythmias, hypertension, diarrheas, hyperplasia, etc [12]. Hence, there is always a need to synthesize novel potential therapeutic agents devoid of side effects and provide ample gastric protection and therapy. The current study is one such effort where we designed, synthesized, and characterized thiazolidine-containing benzothiazole derivatives with a strong potential for rectification of underlying pathological conditions leading to ulcers. These derivatives were evaluated for their antioxidant, anti-inflammatory, and anti-ulcer potential utilizing DPPH assay, carrageenan-induced paw edema, and ethanol-induced ulcer models.

## Materials and methods

### Chemicals

The chemicals were purchased from Sigma-Aldrich, Merck, and Honeywell. During this study analytical grade 2-aminobenzothiazole (CAS no: 136-95-8), chloro-acetyl chloride (CAS no: 79-04-9), benzene (CAS no: 71-43-2), aqueous sodium bicarbonate (CAS no: 144-55-8), ammonium thiocyanate (CAS no: 1762-95-4), ethanol (CAS no: 64-11-5), sodium acetate (CAS no: 127-09-3), acetic acid (CAS no: 64-19-7), carrageenan (CAS no: 9000-07-1), omeprazole (CAS no: 73590-58-6), indomethacin (CAS no: 53-86-1), xylene (CAS no: 116598-94-8) and 3,3′-Diaminobenzidine (CAS no: 91-95-2) were used. All the reactions were carried out using completely dried and cleaned glassware.

### Methodology

#### Synthesis.

**Fig 1:**
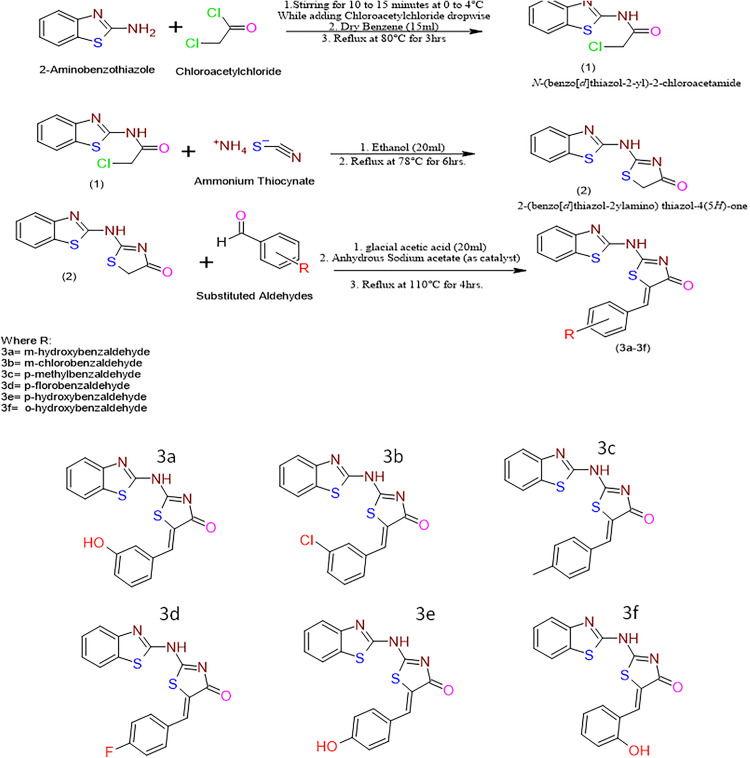
Scheme of synthesis of thiazolidine containing benzothiazole derivatives and structure of synthesized compounds.

In this study, attempts were made to synthesize 2-aminobenzothiazole derivatives (3a-3f) according to the plan of work outlined in the scheme, and the structures of synthesized compounds are given in Fig 1.

#### Purification.

To check the purity of newly synthesized derivatives, TLC was done with Merck TLC Silica gel (60 F) precoated plates using a solvent system (petroleum ether: ethyl acetate 2:1). The spots on TLC plates were visualized with the help of a UV lamp (254nm).

#### Characterization.

All the synthesized compounds were characterized by spectrophotometric analysis with the help of Bruker ALPHA FTIR spectrometer and ^1^H NMR spectra were recorded on Bruker AM300 spectrometer using DMSO-d6 as solvent. The melting points were recorded with Stuart melting point apparatus.

#### Synthesis of *N*-(benzo[*d*]thiazol-2-yl)-2-chloroacetamide.

A solution of 2-aminobenzothiazole (1.50 g, 0.01 mol) in dry benzene (15 mL) was cooled in an ice bath, and chloroacetyl chloride (1.30 mL, 0.0165 mol) was added dropwise over 10–15 minutes with constant stirring, maintaining the reaction temperature between 0–4°C. The mixture was then refluxed at 80°C for 3 hours under a nitrogen atmosphere. Reaction progress was monitored by thin-layer chromatography (TLC) using a chloroform:ethyl acetate (7:3) solvent system. Upon completion, the reaction mixture was cooled and the solvent along with unreacted chloroacetyl chloride was removed under reduced pressure. The resulting residue was washed with cold aqueous sodium bicarbonate (5%) followed by distilled water, and the crude product was recrystallized from ethanol to afford the desired chloroacetamide with a yield of 82% and melting point of 170°C [13] (Fig 1).

#### Synthesis of 2-(benzo[*d*]thiazol-2ylamino) thiazol-4(5*H*)-one.

A mixture of the above chloroacetamide intermediate (0.80 g, 0.0035 mol) and ammonium thiocyanate (0.53 g, 0.007 mol) in absolute ethanol (20 mL) was refluxed at 78°C for 6 hours. TLC using chloroform:methanol (9:1) was used to monitor the progress of the reaction. After completion, the mixture was left overnight at room temperature to allow crystallization. The solid was filtered under vacuum, washed with ethanol-water (1:1), and recrystallized from a 2:1 ethanol:water mixture to obtain the thiazolidinone derivative as a light-yellow crystalline solid (yield 79%, m.p. 214°C) [14] (Fig 1).

### Condensation of thiazolidinone with aldehydes

The final derivatives (BTZ 3a–3f) were synthesized via condensation of the obtained thiazolidinone (0.50 g, 0.002 mol) with appropriate aromatic aldehydes (0.002 mol) in glacial acetic acid (20 mL) using anhydrous sodium acetate (0.33 g, 0.004 mol) as a catalyst. The reaction mixtures were refluxed at 110°C for 4 hours. TLC (ethyl acetate:n-hexane, 6:4) confirmed the completion of each reaction. After cooling to room temperature, the mixtures were poured into ice-cold water (100 mL) with vigorous stirring to precipitate the products. The solids were collected by vacuum filtration, washed with distilled water, and recrystallized from ethanol to yield pure compounds. Yields for the final derivatives ranged from 79% to 86%. The synthesized compounds were confirmed through physical characterization, including melting point, TLC, and spectral analysis (IR, ^1^H-NMR, and ^13^CNMR). Following are the details.

#### (Z)-2-(benzo[*d*]thiazol-2-ylamino)-5-(3-hydroxybenzylidene) thiazol-4(*5H*)-one (3a).

C_17_H_11_N O_2_S_2_; yield 79%; mol wt. 353.41; m.p 212°C; FTIR cm ^−1^ (N-H) 3317, (C-S) 1149, Aromatic (C-H) 2919, Amide (C = O) 1684, ^1^H-NMR (300 MHz, DMSO-d6, δ ppm): 7.34–8.18 (m, 8H, Ar-H), 7.23 (s, 1H, Arylidene-H), 4.12 (s, 1H, NH), 5.31 (s, 1H, OH).

#### (Z)-2-(benzo[*d*]thiazol-2-ylamino)-5-(3-chlorobenzylidene) thiazol-4(*5H*)-one (3b).

C_17_H_10_ClN_3_OS_2_; yield 84.4%; mol wt. 371.86; m.p 210°C; FTIR cm ^−1^ (N-H) 3307, (C-S) 1154, Aromatic (C-H) 3039, Amide (C = O) 1682, ^1^H-NMR (300 MHz, DMSO-d6, δ ppm): 7.33–7.88 (m, 8H, Ar-H), 7.27 (s, 1H, Arylidene-H), 4.04 (s, 1H, NH). 13 C NMR (75 MHz, CDCl_3_, δ ppm): 126.6 (1C), 127.8 (2C), 128.7 (1C), 128.8 (1C), 129.3 (2C), 131.2 (2C), 133.7 (1C), 137.2 (1C), 137.8 (1C), 158.9 (1C), 163.3 (1C), 167.8 (1C), 174.5 (1C)

#### (Z)-2-(benzo[*d*]thiazol-2-ylamino)-5-(4-methylbenzylidene) thiazol-4(*5H*)-one (3c).

C_18_H_13_N_3_OS_2_; yield 81.7%; mol wt. 351.45; m.p 220°C FTIR cm −1 (N-H) 3300, (C-S) 1154, Aromatic (C-H) 3053, Amide (C = O) 1695, ^1^H-NMR (300 MHz, DMSO-d6, δ ppm): 7.41–8.08 (m, 8H, Ar-H), 7.21 (s, 1H, Arylidene-H), 3.98 (s, 1H, NH), 2.34 (s, 3H, CH 3. ^13^C NMR (75 MHz, CDCl 3, δ ppm): δ 29.1 (1C), 126.6 (1C), 127.8 (2C), 128.7 (1C), 128.8 (1C), 129.3 (2C), 130.0 (1C), 130.6 (1C), 132.0 (1C), 136.6 (1C), 152.3 (1C), 158.8 (1C), 162.1 (1C), 168.1 (1C), 179.0 (1C).

#### (Z)-2-(benzo[*d*]thiazol-2-ylamino)-5-(4-fluorobenzylidene) thiazol-4(*5H*)-one (3d).

C_17_H_10_FN_3_OS_2_; yield 79.6%; mol wt. 355.40; m.p 218°C FTIR cm −1 (N-H) 3294, (C-S) 1150, Aromatic (C-H) 3052, Amide (C = O) 1688, ^1^H-NMR (300 MHz, DMSO-d6, δ ppm): 7.30–7.89 (m, 8H, Ar-H), 7.23 (s, 1H, Arylidene-H), 3.93 (s, 1H, NH). ^13^C NMR (75 MHz, CDCl_3_, δ ppm): 121.5 (2C), 121.8 (1C), 124.7 (1C), 126.5 (1C), 130.1 (2C), 130.4 (2C), 134.7 (1C), 141.5 (1C), 167.2 (1C), 168.7 (1C), 169.9 (1C), 170.2 (1C), 172.7 (1C).

#### (Z)-2-(benzo[*d*]thiazol-2-ylamino)-5-(4-hydroxybenzylidene) thiazol-4(*5H*)-one (3e).

C_17_H_11_N_3_O_2_S_2_; yield 83%; mol wt. 353.41; m.p 225°C FTIR cm −1 (N-H) 3315, (C-S) 1148, Aromatic (C-H) 2918, Amide (C = O) 1689, ^1^H-NMR (300 MHz, DMSO-d6, δ ppm): 7.32–7.80 (m, 8H, Ar-H), 7.20 (s, 1H, Arylidene-H), 4.03 (s, 1H, NH), 5.35 (s, 1H, OH).

#### (Z)-2-(benzo[*d*]thiazol-2-ylamino)-5-(2-hydroxybenzylidene)thiazol-4(*5H*)-one (3f).

C_17_H_11_N_3_O_2_S_2_; yield 86%; mol wt. 353.41; m.p 202°C FTIR cm −1 (N-H) 3315, (C-S) 1148, Aromatic (C-H) 2918, Amide (C = O) 1689, ^1^H-NMR (300 MHz, DMSO-d6, δ ppm): 7.42–7.92 (m, 8H, Ar-H), 7.25 (s, 1H, Arylidene-H), 4.12 (s, 1H, NH), 5.30 (s, 1H, OH).

### Evaluation of pharmacological activities

The synthesized compounds were screened for their potential pharmacological activities such as anti-oxidant, anti-inflammatory, and anti-ulcer activity.

### In-vitro anti-oxidant activity

The DPPH (2, 2-Diphenyl-1-picrylhydrazyl) radical scavenging assay was used to quantify the antioxidant activity of the synthesized compounds. A blank solution of DPPH without compound was prepared, methanol served as a negative control, and ascorbic acid was taken as a positive control. The solutions of synthesized compounds in different concentrations µg/ml were added into different tubes and DPPH was added. The tubes were then kept in an incubator at 37°C for 30 min. Because of the anti-oxidant property of the newly synthesized compound, the color of DPPH was changed. A UV/Vis spectrophotometer was used to measure changes in absorbance at 517 nm. Reduction of 2−2,diphenyl-1-picrylhydrazyl absorption at a wavelength of 517nm represents the free radical scavenging ability of the test compounds. The radical scavenging activity was estimated using the following equation [15].


𝐑𝐚𝐝𝐢𝐜𝐚𝐥 𝐬𝐜𝐚𝐯𝐞𝐧𝐠𝐢𝐧𝐠 𝐚𝐜𝐭𝐢𝐯𝐢𝐭𝐲 (%)=[(A0−A1)/A0]×100

Where A0 represents the absorbance of the control (blank, without compound) and A1 represents the absorbance of the compound. Based on the best anti-oxidant properties, compounds were selected for further studies.

### *In-vivo* study design and Pharmacological potential

#### Animals.

Albino mice weighing 25–30 g of either sex were taken from the animal house of Riphah International University, Islamabad. Mice were placed in plastic cages at controlled temperature and given free access to food and water. Experimental procedures used for animal studies were approved by Riphah Institute of Pharmaceutical Sciences Ethical Committee. The researchers were blinded to treatment groups during data collection/analysis, to minimize bias.

### Ethical statement

The animals used in the experimentations were owned by the Riphah Institute of Pharmaceutical Sciences and housed in their animal house. The experiments were carried out according to the ARRIVE guidelines, with the approval of the Research and Ethics Committee of Riphah Institute of Pharmaceutical Sciences, Riphah International University (Reference no: REC/RIPS/2022/06). All animals were anesthetized using a cocktail of xylazine (9 mg/kg) and ketamine (90 mg/kg) intraperitoneally and were euthanized following AVMA guidelines. During this process, in mice, the thumb and index finger on opposite sides at the base of the skull or a rod was pressed to that same site. The other hand was used to seize the base of the tail or the hind limbs, and a sharp pull confirmed the separation of cervical vertebrae from the skull. We have applied all the laboratory procedures to minimize the animal’s suffering, such as heating pad, sterilization and fluid replenishment with normal saline. Animals showing signs of distress and loss of appetite were taken as humane endpoints and animals were sacrificed within one hour of dosing. None of the animals died during the study.

### Anti-inflammatory activity

Carrageenan-induced paw edema assay was performed to evaluate the anti-inflammatory potential of the selected compounds. In this experiment, 30 mice (25-27g) of either sex were randomly divided into five groups, each with six mice. Group I (control), Group II (disease), III and IV (treatment), and Group V (standard). To identify each animal, an indelible pen was used to mark the tails. Animals in the control group received normal saline. In all the groups except group I, paw edema was induced by intra-plantar injection of 0.5% carrageenan (suspension in normal saline). The treatment groups received doses (2 mg/kg) of the test compounds 3d and 3e before 30 minutes of the carrageenan injection. Indomethacin (10 mg/kg) was employed as a standard drug. Baseline readings were taken before inducing edema. After 1 hour of carrageenan injection paw, volume was measured hourly for a period of 6 hours by using a plethysmometer. After the last reading, animals were sacrificed and paws were removed [16].

### Anti-ulcer activity

36 Mice of either sex, weighing between 25 and 30 g, were placed into groups at random (n = 6/group) and maintained fasting for 24 hours. Group I received a 10 mL/kg saline solution. Group II received oral dosage of ethanol (1 mL/100 g) to induce gastric ulcer. Compounds 3b, 3d, and 3e (5 and 10 mg/kg) were given orally to Groups III, IV, and V, one hour before ethanol administration (1 mL/100 g). Omeprazole 20 mg/kg was orally administered to Group VI as a standard treatment, one hour before ethanol administration (1 mL/100 g). After one hour of the last treatment, the animals were sacrificed. Before computing the lesion index, stomachs were removed and washed in normal saline. The ulcer index uses each lesion’s mean ulcer score. Each lesion was measured in millimeter which were then added to determine the ulcer index (UI). I% is used to measure gastroprotective activity [6].


% 𝐈=(𝐔𝐒𝐜−𝐔𝐒𝐭)×100/𝐔𝐒𝐜

Where USc = ulcer surface area of control and USt = ulcer surface area of test animal.

### Tissue preparation for biochemical analysis

Both paw and gastric tissue samples were homogenized in 0.1 M sodium phosphate buffer containing PMSF as a protease inhibitor. Samples were centrifuged for 10 minutes at 4000 X speed and the supernatant was collected. This supernatant was then processed for further biochemical analysis.

### Determination of oxidative stress markers

The supernatant was removed after homogenizing the isolated mice’s stomach tissues and paw tissues by centrifuging at 1,500 rpm for 30 minutes. Catalase, glutathione (GSH), lipid peroxidation (LPO), and glutathione-S-transferase activity (GST) were measured in the supernatants. GSH was measured using 2-nitro-5-thiobenzoic acid, a yellowish byproduct of GSH and DTNP oxidation [9]. With a microplate reader, 412 nm absorbance was determined. GSH is measured in µmoles per mg of protein. The formation of CDNB was used for measuring GST activity at an absorbance of 340 nm. The rate of CDNB conjugate formation per minute per milligram of protein is the unit of measure for GST activity [17]. The Degradation of H2O2 measured catalase activity. 240 nm absorbance was measured using a microplate reader. The catalase activity was measured in moles of H_2_O_2_/min/mg protein [10]. Malondialdehyde, a by-product of LPO is used to determine the extent of lipid peroxidation of the tissue. To measure absorbance at 532 nm, a microplate reader was employed. LPO was measured in units of nmoles/min/mg protein TBARS [18]. For SOD (superoxide dismutase), an assay was performed according to previously established protocols. Superoxide radicals generated by xanthine and xanthine oxidase and reaction with nitro blue tetrazolium produces formazan dye. Superoxide dismutase activity was estimated by inhibition of the aforementioned reaction and inhibition determined at 560 nm and expressed as millimole per minute per milligram tissue (mmol/min/mg/tissue) [19].

### Hematoxylin and eosin staining (H&E staining)

In each group, six mice were utilized for the morphological analysis of both paw and gastric tissues (n = 6/group). 10% paraformaldehyde was used to fix both the paw and gastric tissues and kept them there until further investigation. Hematoxylin and eosin (H&E) staining was performed after tissue slices were made using a rotary microtome [20]. Stained tissue sections were examined under a microscope, and printouts were taken.

### Immunohistochemical analysis

The gastric and paw tissues were stained immunohistochemically. Three separate absolute xylene solutions were used to deparaffinize tissue sections mounted on slides and rehydrated with ethyl alcohol in a range of concentrations (from 100% [absolute] to 70%). The slides were then kept in 0.01 M phosphate-buffered saline (PBS) for 10 min after being rinsed with distilled water. The slides were then treated with appropriate biotinylated secondary antibodies for 2 hours after the antigen retrieval step, and 1 hour with Avidin-biotin complex (ABC) reagents (Santa Cruz Biotechnology, United States) at room temperature. The sections were washed with PBS, stained with 3,3′-Diaminobenzidine (DAB) solution, dehydrated in graded ethanol solutions (70, 95, and 100%), fixed in xylene, cover-slipped with a mounting medium, and then allowed to dry naturally. The findings were examined with a high-end digital photo microscopy system attached to a light microscope (Olympus, Japan). Immunohistochemical TIF pictures were captured using a light microscope (5 photos per plate). Phosphorylated nuclear factor-kappa β (SC-271908 Santa Cruz Biotechnology, Dallas, Tx, United States), tumor necrosis factor α (SC-52B83 Santa Cruz Biotechnology, Dallas, Tx, United States), and COX-2 (SC-514489 Santa Cruz Biotechnology, Dallas, Tx, United States) antibodies were quantified using ImageJ software [11].

### Enzyme-linked immunosorbent assay (ELISA)

The p-NFkB, TNF-α, and COX-2 expression were measured by ELISA using mouse-ELISA kit TNF-α (Catalog No: PRS-30651Ra), p-NFkB(Catalog No: PRS-20640Mo) and COX-2 (Catalog NO. PRS-30205Ra) according to manufacturer’s instructions. Paw and stomach tissues of mice (50 mg) were homogenized in PBS using SilentCrusher M (Heidolph) at 15000 rpm. Then, the sample was centrifuged at 4000 rpm for 30 minutes and the supernatant was collected. Protein content in each group was determined by the BCA method. Briefly, using a 96-well plate, protein samples were loaded to determine the concentration of p-NFkB, COX-2, and TNF-α. Absorbance values were measured through a microplate reader (BioTek ELx808). All steps were performed in triplicate.

### *Insilico* pharmacokinetic profiling

In silico pharmacokinetic profiling of the synthesized chalcone derivatives was carried out using the SwissADME web tool to evaluate key ADME parameters including gastrointestinal absorption, blood-brain barrier permeability, and drug-likeness based on Lipinski’s rule of five [21]. These predictions facilitated early-stage screening of compounds for drug-likeness and bioavailability.

### Molecualr docking

For molecular docking, the 3D crystal structure of the target protein was retrieved from the Protein Data Bank (PDB), and docking simulations were performed using PyRx 0.8. The most stable conformers were selected based on binding energy scores and analyzed for key interactions using Discovery Studio Visualizer [22]. This computational approach helped predict the binding affinity and orientation of ligands within the active site of the receptor.

### Statistical analysis

All the data is expressed as ± standard error of mean (SEM) and mean ± standard deviation (SD). Image J software (NIH, USA) was utilized for morphological data while other data was analyzed using one-way ANOVA followed by post-hoc Tukey’s test using Graph Pad program (GraphPAD, SanDiego, CA-USA). *P* < 0.05 was regarded as significant. # denotes analysis with respect to saline while * denotes analysis with respect to the disease group.

## Results

### *In-vitro* Anti-oxidant activity analysis of synthesized compounds

In-vitro analysis of all the synthesized derivatives is demonstrated in Table 1. Among all the synthesized derivatives the highest antioxidant activity was reported by compound 3e with 92.1% inhibition and an IC_50_ value of 5.64 µg/ml which is comparable to the positive control ascorbic acid. Two other compounds 3d and 3b also exhibited comparatively higher antioxidant activity with % inhibition of 90.4% and 82.43% and IC_50_ value of 6.6 µg/ml and 7.2 µg/ml respectively. 3a, 3c and 3f exhibited lower anti-oxidant potential. Only compounds with high anti-oxidant ability were selected for further in-vivo analysis.

**Table 1:** Antioxidant activity and IC_50_ values of the synthesized derivatives.

Samples	%Inhibition	IC_50_ (µg/ml)
3a	76.78	10.9
3b	82.43	7.2
3c	74.56	8.2
3d	90.4	6.6
3e	92.1	5.6
3f	61.4	13.4
Ascorbic acid	94	4.3

### Pharmacological analysis of compounds with high anti-oxidant potential

Based on the free radical scavenging ability of all synthesized derivatives, only 3b, 3d, and 3e were further analyzed for their pharmacological potentials in carrageenan-induced paw edema and gastric ulcer.

### Synthesized benzothiazoles possess anti-inflammatory potential and improved histological alterations in carrageenan-induced paw edema

**Fig 2:**
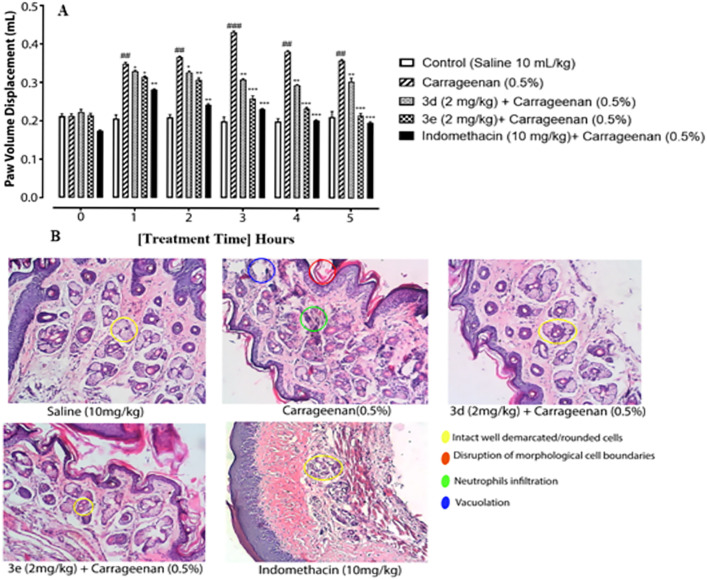
Synthesized benzothiazoles possess anti-inflammatory potential and improved histological alterations in carrageenan-induced paw edema A) Anti-inflammatory effect of compounds (3d, 3e) and indomethacin in carrageenan-induced paw edema in mice. Each bar represents paw volume displacement after 1, 2, 3, 4, and 5h of treatment. The data was analyzed by ANOVA followed by *post-hoc* Tukey’s test. ^###^p < 0.001 vs. saline group, ^**^p < 0.01, ^***^p < 0.001 vs. carrageenan group (n = 6/group), B) Histopathological alterations as shown by H&E staining (10x, scale bar 50µm n = 6/group).

The test compounds 3d and 3e, selected based on their high radical scavenging ability, were analyzed for their anti-inflammatory potential in carrageenan-induced mice paw edema and the histological changes it imparted (Fig 2A, 2B). Carrageenan induced substantial inflammation after injection (Fig 2A; ###p < 0.001) and 3d and 3e at the dose of 2 mg/kg exhibited an anti-inflammatory effect that became significant two hours after carrageenan injection and sustained all along the experiment (Fig 2A; ^*^p < 0.05, ^**^p < 0.01). After 5 hours, compound 3d and 3e both demonstrated significant paw volume displacement (Fig 2A; ^***^p < 0.001), comparable to that of indomethacin (Fig 2A; ^***^p < 0.001)

Paw tissues were also examined for histopathological alterations through H&E staining. Upon microscopic analysis, it was observed that the saline group showed perfectly normal well-demarcated, rounded cells free of any pathological alterations and lesions (Fig 2B). The Edematous mice paw tissues, however, manifested severe cellular disruptions including vacuolation, neutrophil infiltration, and deformed and damaged morphological cell boundaries. Upon treatment with 3d, 3e, and indomethacin, the paw tissues exhibited signs of improvement in both cellular morphology and structure, while cellular regeneration and repair can be observed in Fig 2B.

### Benzothiazole derivatives ameliorated exaggerated inflammatory mediators in carrageenan-induced rat’s paw edema

**Fig 3:**
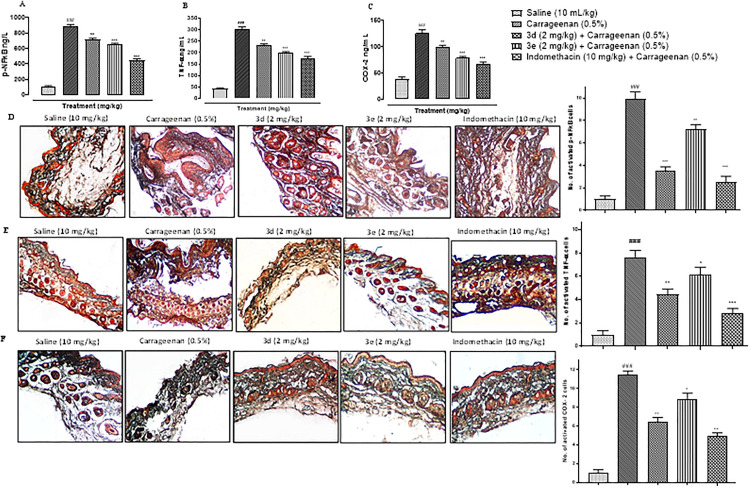
Ameliorative potential of compound 3d, 3e and indomethacin on inflammatory markers (A) NFκB expression as quantified by ELISA (D) Immunohistochemistry results for p-NFκB expression in mouse paw following edema (B) TNF-α expression in carrageenan treated mice using ELISA (E) immunohistochemistry results for TNF-α in mouse paw following edema (C) COX-2 expression in carrageenan treated mice using ELISA (F) immunohistochemistry results for COX-2 in mouse paw edema. The data are expressed as mean ± SEM, n = 6/group. ^###^p < 0.001 vs saline. ^*^p < 0.05 ^**^p < 0.01, ^***^p < 0.001 vs. carrageenan group. Scale bar 50µm, 10x.

The paw tissues were subjected to immunohistochemical analysis and ELISA to estimate the degree of expression of pro-inflammatory and inflammatory markers such as p-NFkB, TNFα, and COX-2. An excessive elevation in the expression of P-NFkB, TNFα, and COX-2 can be seen in disease groups (Fig 3A–3F; ^###^p < 0.001 vs. Saline group). A significant amelioration in P-NFkB expression was observed after treatment with BTZ derivatives 3d and 3e, comparable to that of indomethacin (Fig 3A, 3D; ^**^p < 0.01; ^***^p < 0.001 vs. Carrageenan group). A similar trend was observed for TNF-α after treatment with 3d and 3e (Fig 3B, 3E; ^*^p < 0.05, ^**^p < 0.01, ^***^p < 0.001 vs. Carrageenan group). Moreover, COX-2 expression was also exaggerated due to inflammation which was then mitigated by administration of 3d and 3e (Fig 3C, 3F; ^*^p < 0.05, ^**^p < 0.01, ^***^p < 0.001 vs. Carrageenan group)

### Compounds 3d and 3e restored anti-oxidant markers in paw edema

**Fig 4:**
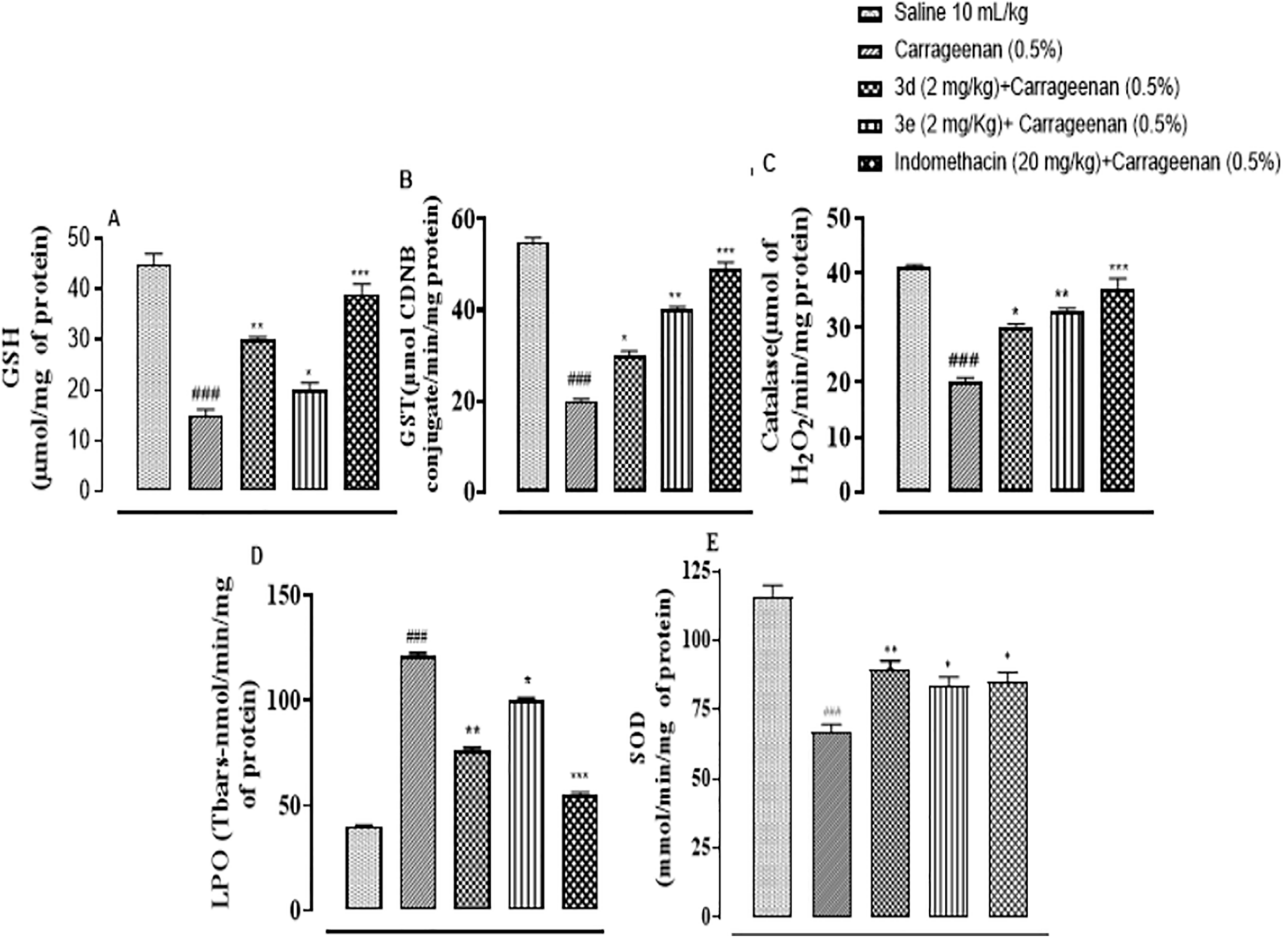
Compounds 3d and 3e restored antioxidant markers in paw edema (A) GSH, (B) GST, (C) CAT, (D) LPO and (E) SOD in carrageenan-induced paw edema in mice paw tissues. Values expressed as mean ± SEM. One way ANOVA with *post-hoc* Tukey’s test. ^###^p < 0.001 vs, saline group, ^*^p < 0.05, ^**^p< 0.01, ^***^p < 0.001 vs, carrageenan group. n = 6/group.

To determine the degree of free radical generation due to paw edema, the activity of GSH, GST, catalase, LPO and SOD was carried out. All the antioxidants GST, GSH, catalase and SOD were significantly diminished in edematous tissue with an excessive elevation of LPO (Fig 4A–4E; ^###^p < 0.001 vs. Saline group). Upon treatment with 3d, 3e, and indomethacin, the levels of antioxidants were restored whereas considerable reduction in lipid peroxidation was reported (Fig 4A–4E; ^*^p < 0.05, ^**^p < 0.01, ^***^p < 0.001 vs. Carrageenan group).

### Synthesized benzothiazoles improved mucosal damages and histological alterations in ethanol-induced gastric mucosal injury

**Fig 5:**
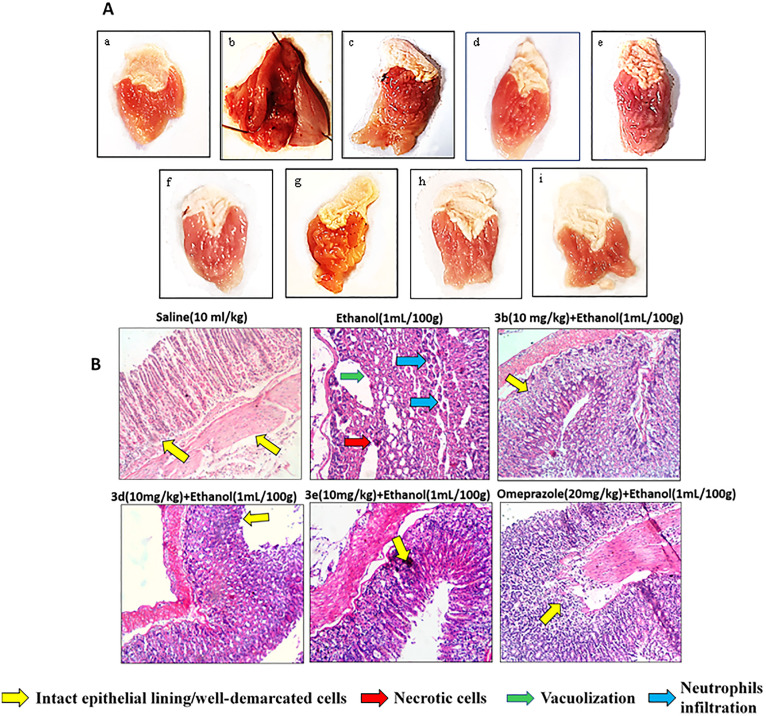
Synthesized benzothiazoles improved mucosal injury and histological damages in ethanol-induced gastric mucosal injury (A) Gross appearance of the gastric mucosa of mice pretreated with (a) saline (10mL/kg), (b) ethanol (1 mL/100g) severe injuries are seen, as ethanol produced excessive necrosis of gastric mucosa, pretreated with (c) 3b (5 mg/kg), (d) 3b (10 mg/kg), (e) 3d (5 mg/kg), (f) 3d (10 mg/kg), (g) 3e (5 mg/kg), (h) 3e (10 mg/kg), (i) omeprazole (20 mg/kg). (B) Histopathological slides showing the effect of compound 3b, 3d, 3e, and omeprazole in ethanol-treated mice gastric tissues using hematoxylin and eosin staining histopathological technique. Bar 50 µm, magnification 10x. n = 6/group.

After drug treatment, macroscopic evaluation of gastric mucosa indicated an alleviation in mucosal lesions as measured by lesion index (Table 2). In the normal saline group, intact mucosa could be seen which was ulcerated with patchy hemorrhagic sections after ethanol administration (Table 2, ^###^p < 0.001 vs. Saline group; Fig 5A). A gross examination of gastric mucosa revealed both mucosal and submucosal lesions and lacerations in the ethanol group. Erosions and hypertrophic alterations were observed (Fig 5A). Pretreatment with 3b, 3d, and 3e remarkably reduced visible hemorrhagic and erotic lesions induced by ethanol in mice’s stomach. BTZ derivatives also improved the histological structure of the surface epithelium and confirmed its protective role in ethanol-induced mucosal degeneration and ulcers (Fig 5A). Furthermore, omeprazole indicated a 90% improvement in mucosa which is well documented (Table 2; ^***^p < 0.001 vs. Ethanol group)

**Table 2:** Effect of compounds and omeprazole against ethanol-induced ulcer in mice.

Treatment (mg/kg)	Ulcer Index	%Inhibition
Saline (10mL/kg)	0.0 ± 0.2	100
Ethanol (1mL/100 g)	10.2 ± 0.2^###^	0
3b (5 mg/kg) + Ethanol (1 mL/100g)	7.5 ± 0.19^*^	26.47
3b (10 mg/kg) + Ethanol (1 mL/100g)	3 ± 0.2^***^	70.58
3d (5 mg/kg) + Ethanol (1 mL/100g)	4.5 ± 0.15^**^	55.8
3d (10 mg/kg) + Ethanol (1 mL/100g)	2.5 ± 0.13^***^	75.49
3e (5 mg/kg) + Ethanol (1 mL/100g)	7 ± 0.22^*^	31.37
3e (10 mg/kg) + Ethanol (1 mL/100g)	2 ± 0.19^***^	80.39
Omeprazole (20 mg/kg) + Ethanol (1 mL/100g)	1 ± 0.14^***^	90.1

Values expressed as mean ± SEM (n = 6/group). One-way analysis of variance (ANOVA) followed by *post-hoc* Tukey’s test. ^###^*P <* 0.001 vs*.* saline group*,* ^*^p < 0.05, ^**^p < 0.01, ^***^p *<* 0.001 vs. Ethanol group.

Upon histopathological analysis through H&E staining, the saline group expressed normal stomach tissues with architecture free of any pathological alteration (Fig 5B). Mice gastric tissues ulcerated by ethanol caused severe stomach tissue erosion, vacuolation, and destruction of morphological cell boundaries along with gastric lesions. The gastric tissues of mice treated with 3b, 3d, 3e and omeprazole exhibited cellular regeneration with reduced gastric erosion and lesions, and improved gastric mucosa (Fig 5B).

### Compounds 3b, 3d, and 3e mitigated inflammatory mediators after gastric mucosal injury

**Fig 6:**
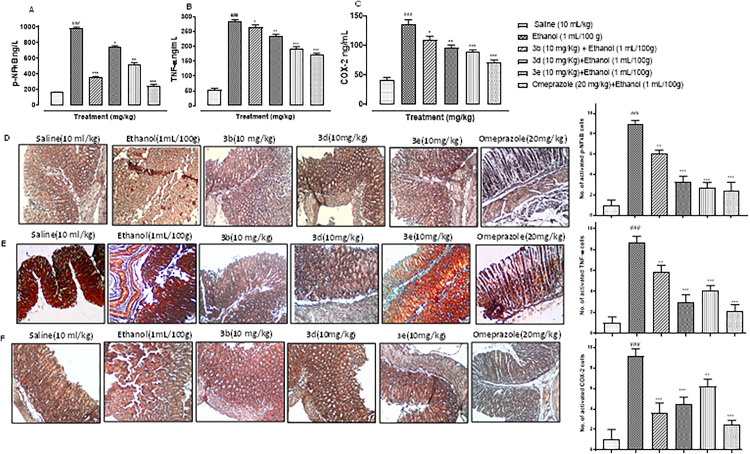
Compounds 3b, 3d, and 3e mitigated inflammatory mediators after gastric mucosal injury (A) NFκB expression as quantified by ELISA (D) Immunohistochemistry results for p-NFκB expression in mouse gastric ulcer (B) TNF-α expression in ethanol-treated mice using ELISA (E) Immunohistochemistry results for TNF-α in mouse gastric ulcer (C) COX-2 expression in ethanol-treated mice using ELISA (F) Immunohistochemistry results for COX-2 in mouse gastric ulcer. The data are expressed as mean ± SEM, n = 6/group. ^###^p < 0.001 vs. saline.^***^p < 0.001, ^**^p < 0.01, ^*^p < 0.05 vs ethanol group. Scale bar 50µm, 10x.

IHC analysis and ELISA of stomach tissues indicated an excessive overexpression of proinflammatory and inflammatory markers COX-2, p-NFκB, and TNF-α after ethanol administration (Fig 6A–6F; ^###^p < 0.001 vs. Saline group). A significant amelioration in p-P-NFkB expression was observed after treatment with BTZ derivatives 3b, 3d, and 3e as quantified by both ELISA and Immunohistochemistry (Fig 6A, 6D; ^*^p < 0.05, ^**^p < 0.01, ^***^p < 0.001 vs. Ethanol group). A similar trend was observed for TNF-α (Fig 6B, 6E; ^*^p < 0.05, ^**^p < 0.01, ^***^p < 0.001 vs. Ethanol group) and COX-2 (Fig 6C, 6F; ^*^p < 0.05, ^**^p < 0.01, ^***^p < 0.001 vs. Ethanol group). The derivatives thus significantly mitigated their release and reduced their expression prominently, indicating an anti-inflammatory potential of these newly synthesized derivatives in gastric ulcers, hence restoring gastric integrity.

### Compounds 3b, 3d, and 3e possess anti-oxidant potential as seen in gastric mucosal injury

**Fig 7:**
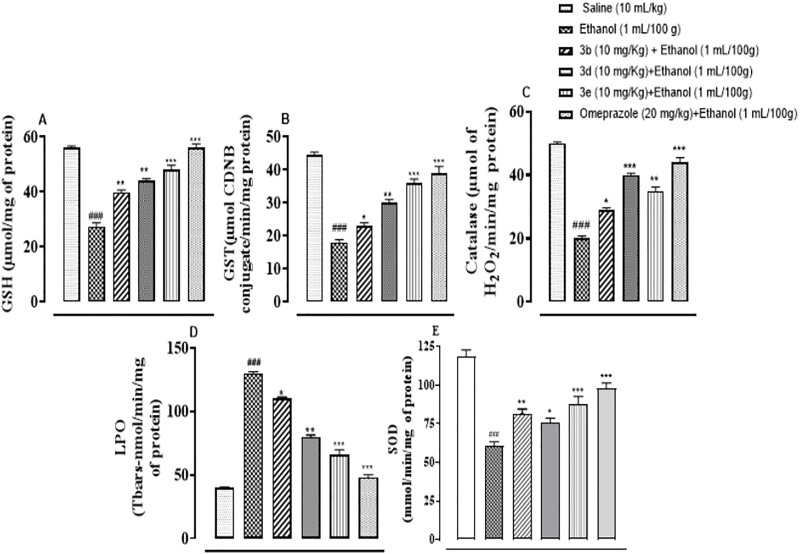
Treatment groups (3b, 3d, 3e) demonstrated antioxidant potential against oxidative stress (A) GSH, (B) GST, (C) CAT, (D) LPO and (E) SOD in ethanol-induced ulcers in mice stomach tissues. Values expressed as mean ± SEM (n = 6/group). One-way ANOVA with *post-hoc* Tukey’s test. ^###^p < 0.001 vs saline group, ^*^p < 0.05, ^**^p< 0.01, ^***^p < 0.001 vs ethanol group.

The antioxidant profile of gastric mucosal tissue was carried out post-treatment. All the antioxidant levels of GSH, GST, catalase and SOD were significantly diminished while an elevated LPO level was observed after ethanol administration (Fig 7A–7E, ^###^p < 0.001). However, the compounds 3b, 3d, 3e, and omeprazole restored GSH, GST, catalase and SOD levels, considerably reducing lipid peroxidation (Fig 7A–7E).

### Insilico pharmacokinetic study

#### Absorption and distribution.

In silico pharmacokinetic evaluation using SwissADME revealed that all six synthesized benzothiazole-thiazolone derivatives (3a–3f) exhibited high gastrointestinal (GI) absorption, which is an encouraging indicator for oral bioavailability. Compounds such as 3a, 3c, and 3f showed optimal absorption characteristics, which can be attributed to a favorable balance between lipophilicity and polar surface area. Solubility estimates across different predictive models (ESOL, Ali, and Silicos-IT) categorized most derivatives as moderately soluble, with 3f particularly demonstrating improved aqueous solubility. Regarding distribution, only 3b, bearing a chloro-substitution, was predicted to penetrate the blood-brain barrier (BBB)—a property potentially linked to increased lipophilicity. The remaining analogues were deemed non-BBB permeant, suggesting they are less likely to exhibit central nervous system activity. Additionally, skin permeability, as estimated by log Kp values (ranging from –5.19 to –6.11 cm/s), suggested that these molecules are poorly absorbed through the dermal route, reinforcing their systemic application potential.

#### Metabolism.

The metabolic interaction profile, particularly regarding cytochrome P450 (CYP) inhibition, was also predicted. Most derivatives showed no inhibitory effect on key isoforms such as CYP2C9, CYP2D6, and CYP3A4, indicating a reduced risk of adverse metabolic interactions. Interestingly, compounds 3d and 3f showed a potential to inhibit CYP1A2 and CYP2C19, which could be influenced by the presence of specific substituents like the fluorine atom in 3d and ortho-hydroxy group in 3f. These functional groups may enhance binding affinity to CYP enzymes, possibly affecting metabolic clearance. Overall, the absence of broad-spectrum CYP inhibition in most molecules supports their metabolic safety, particularly in derivatives like 3a, 3c, and 3e, which may offer a more stable pharmacokinetic profile with lower risk of hepatic liabilities.

#### Excretion.

Although SwissADME does not directly predict renal or biliary excretion, certain molecular descriptors offer indirect insight. The low log Kp values, consistent across all derivatives, suggest minimal passive diffusion through the skin, and thereby low potential for dermal elimination. Moreover, the compounds’ moderate molecular weights (ranging between 351 and 372 Da) and compliance with Lipinski’s rule of five indicate a favorable size and polarity range for renal excretion following hepatic metabolism. The synthetic accessibility scores (approximately 3.4 to 3.6) reflect moderate ease of chemical synthesis and potential compatibility with metabolic transformation and clearance mechanisms. Collectively, the ADME profiling suggests that the synthesized scaffolds—especially 3a, 3d, and 3f—demonstrate a pharmacokinetically favorable foundation for further optimization and preclinical development.

### Molecular docking analysis

Molecular docking studies were conducted to evaluate the binding affinity of the synthesized benzothiazole-based thiazolone derivatives (3a–3f) with the active site of cyclooxygenase-2 (COX-2, PDB ID: 1PXX) and nuclear factor Ƙb (NFκB, **PDB ID: 1IKN)**. The binding energy values ranged from −5.8 to −8.3 kcal/mol for COX-2 and −7.3 to −8.6 for NFκB, indicating moderate to strong interactions (Table 3). The binding interactions of compunds with the respective targets are shown in S1 and S2 Figs.

**Table 3:** Binding affinities of newly synthesized derivatives.

Compounds	Target	
	COX-2 (PDB ID:1pxx)	NFκB (PDB ID 1IKN)
3a	−7	−8.2
3b	−6.9	−8.4
3c	−5.9	−7.3
3d	−7.4	−8.4
3e	−5.8	−8.6
3f	−8.3	−8.4

### Structure–Activity Relationship (SAR)

The antioxidant activity of the synthesized benzothiazole-thiazolidinone derivatives was strongly influenced by the electronic nature and position of substituents on the arylidene moiety. Compound 3e, bearing a para-hydroxy group, exhibited the highest DPPH radical scavenging activity (92.1% inhibition, IC_50_ = 5.6 µg/mL), statistically comparable to ascorbic acid (94%, IC_50_ = 4.3 µg/mL; *p < 0.001 vs. 3f). In contrast, its ortho-isomer 3f showed significantly lower antioxidant activity (61.4% inhibition, IC_50_ = 13.4 µg/mL), suggesting that steric hindrance and intramolecular hydrogen bonding at the ortho-position may reduce free radical accessibility. Halogenated analogues 3d (4-fluoro) and 3b (3-chloro) also demonstrated strong antioxidant activity (90.4% and 82.4% inhibition, IC_50_ = 6.6 and 7.2 µg/mL respectively; p < 0.01 vs. 3a and 3c), attributed to their electron-withdrawing nature and ability to stabilize electron density on the aromatic ring. The methyl-substituted 3c showed moderate antioxidant potential (74.56%, IC_50_ = 8.2 µg/mL), consistent with the limited donating effect of alkyl groups.

Similar structural influences were observed in anti-inflammatory and anti-ulcer assays. In carrageenan-induced paw edema, compounds 3e and 3d significantly reduced paw swelling at 2 mg/kg, with maximal inhibition observed at 5 hours (p < 0.001 vs. carrageenan group), comparable to indomethacin. Immunohistochemistry and ELISA confirmed reduced expression of COX-2, TNF-α, and p-NFκB in treated groups (**p < 0.01 to *p < 0.001 vs. disease control). In the ethanol-induced ulcer model, 3e at 10 mg/kg significantly reduced ulcer index to 2.0 ± 0.19, achieving 80.39% inhibition (p < 0.001 vs. ethanol control), whereas 3b and 3d showed ulcer indices of 3.0 ± 0.2 and 2.5 ± 0.13, corresponding to 70.58% and 75.49% inhibition, respectively. These findings suggest that para-OH and halogen substituents enhance anti-inflammatory and anti-ulcer efficacy, likely through synergistic antioxidant, anti-inflammatory, and mucosal protective mechanisms. The data clearly support that substituent position and electronic characteristics are key determinants of biological activity across the synthesized series.

## Discussion

Benzothiazole core has been a moiety of interest for many years due to its potent pharmacological activities [23]. Benzothiazoles belong to a heterocyclic group of compounds and have been identified as having a wide spectrum of pharmacological activities ranging from antioxidant to anticancer. Many benzothiazole derivatives have been reported to possess activities as antitumor, anti-inflammatory [24], antidiabetic, anti-rheumatic, antidepressant, antibacterial [25,26] and many others. Many BTZ derivatives have shown remarkable potential against various tumor cell lines such as HeLA, HepG2, ovarian tumors etc. [27]. Based on these reported data, we designed, synthesized, and characterized six thiazolidinone-containing benzothiazole derivatives. These were then assessed by spectroscopic analysis including FT-IR, HNMR, and TLC screening. Preliminary screening of all the new derivatives was done by *in vitro* DPPH free radical assay, to avoid unnecessary loss of animals. All the compounds exhibited variable anti-oxidant ability and the most potent compounds were further analyzed for their anti-inflammatory activity in the inflammation model of paw edema and gastric ulcer. The selected BTZ derivatives were then subjected to the assessment of their anti-inflammatory and anti-ulcer potential in mice models of carrageenan-induced rat’s paw edema and ethanol-induced gastric ulcer respectively. The in vivo results demonstrated that BTZ derivatives remarkably reduced carrageenan and ethanol-induced inflammation, oxidative stress, and cellular degeneration which has already been reported for many benzothiazole derivatives in numerous studies.

Oxidative stress and ROS generation have been identified as a pathological basis of many degenerative diseases [8]. Oxidative stress is directly linked to inflammation in many neuronal [9] and gastric models of ulcer [6]. We evaluated the oxidative profile of these derivatives after administration in both the carrageenan-induced edema model and gastric ulcer. As reported earlier, the levels of all antioxidants were extremely reduced in both these models, whereas after administration, BTZ derivatives successfully restored these levels significantly. Since oxidative stress induces inflammation, we then evaluated the anti-inflammatory potential of these derivatives through the carrageenan-induced paw edema model which has been widely employed in testing the anti-inflammatory effect of many drugs [28]. Carrageenan initiates the acute phase of inflammation which is biphasic. Initially, there is a release of histamine, kinins, and serotonins after injection of carrageenan [29], which then proceeds to the release of prostaglandins in a few hours [30]. These prostaglandins are the major culprits in inducing inflammation by releasing inflammatory mediators [31] and this phase is most sensitive to anti-inflammatory drugs. Our results also exhibited an excessive release of inflammatory mediators which were mitigated by the administration of BTZ derivatives thus inducing anti-inflammatory effects.

We also evaluated the role of these BTZ derivatives in the ethanol-induced gastric ulcer model. This model is associated with excessive production and release of free radicals, causing oxidation of cellular components and depletion of the anti-oxidant system of the body [7]. Moreover, ethanol also elevates the release of inflammatory mediators, promoting neutrophil infiltration and mucosal damage which has been observed as a direct consequence of oxidative damage [32]. Our study is also in line with the previous research and demonstrated diminished levels of all antioxidants CAT, GST, and GSH after ethanol administration. This effect was ameliorated after treatment with the BTZ derivatives, revealing their strong antioxidant potential, which may be attributed to not only the BTZ moiety but may also be enhanced due to the substitutions of the BTZ ring. Furthermore, an exorbitant overexpression of the inflammatory markers p-NFkB, TNF-α, and COX-2 was observed post-ethanol administration, as reported by previous data [11], which was mitigated after BTZ derivatives, thus confirming that excessive inflammation aggravates gastric ulcers and the protective role of these derivatives. However, gastric ulcer induced by ethanol does not fully replicate chronic ulcer pathophysiology, and hence utilization of H-Pylori and NSAIDS to induce ulcer may provide a thorough insight into the anti-ulcer potential of these derivatives.

## Conclusion

2-amino benzothiazole derivatives are important in medicinal chemistry and have a wide range of pharmacological activities. This research aimed to investigate the pharmacological role of newly synthesized and characterized benzothiazole derivatives in paw edema and gastric ulcer disease. Among the synthesized derivatives, compounds 3b, 3d and 3e demonstrated maximum antioxidant activity which were then proceeded to assess their anti-inflammatory potential in carrageenan-induced paw edema and gastric ulcer models. Compounds, 3b, 3d and 3e exhibited potent anti-inflammatory as well as anti-ulcer activity. They can be used as lead compounds for further drug discovery, however, their underlying mechanisms and effects on other components of inflammatory systems and ulcer pathology still need to be explored.

## Supporting information

S1 Fig 2D interactions of compound 3a (A), 3b (B), 3c (C), 3d (D), 3e (E) and 3f (F) with COX-2 respectively drawn through Discovery Studio Client version 2021. (PDF)

S2 Fig 2D interactions of compound 3a (A), 3b (B), 3c (C), 3d (D), 3e (E) and 3f (F) with NFκB respectively drawn through Discovery Studio Client version 2021. (PDF)

S1 File Proton NMR and Carbon NMR of compounds. (PDF)

S2 File Raw data for SWISSADME. (CSV)
