# Supplementary material for: Synthesis of new benzothiazole derivatives with in-depth In-vitro, In-vivo anti-oxidant, anti-inflammatory and anti-ulcer activities
Source: PLoS One. 2026 Jan 30;21(1):e0337639. doi: 10.1371/journal.pone.0337639 (PMC12857961; doi:10.1371/journal.pone.0337639)
Supplement: S1 Fig — (PDF) [file pone.0337639.s001.pdf]

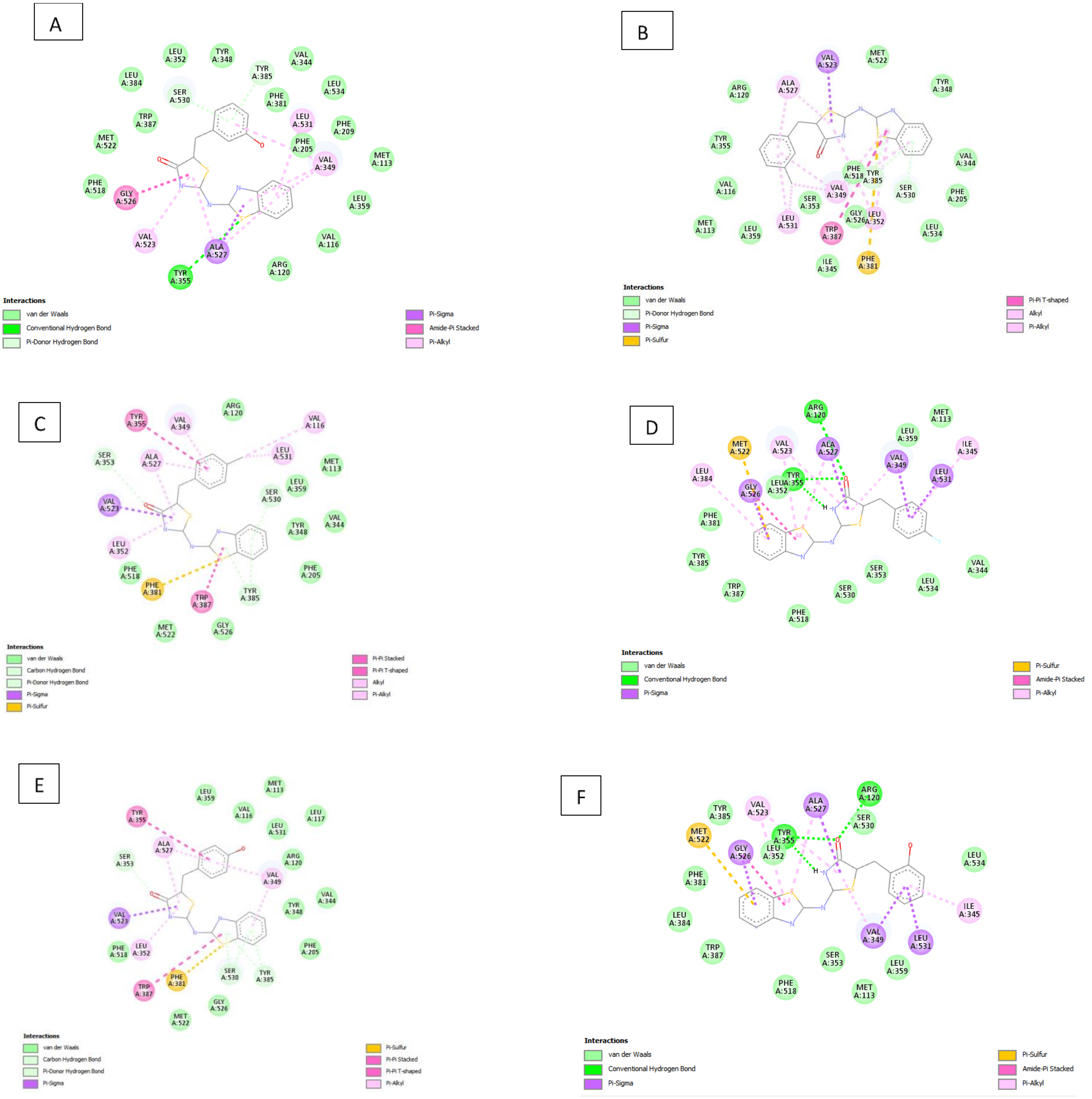

Figure S1. 2D interactions of compound 3a (A), 3b (B), 3c (C), 3d (D), 3e (E) and 3f (F) with COX-2 respectively drawn through Discovery Studio Client version 2021.
