# Supplementary material for: Synthesis of new benzothiazole derivatives with in-depth In-vitro, In-vivo anti-oxidant, anti-inflammatory and anti-ulcer activities
Source: PLoS One. 2026 Jan 30;21(1):e0337639. doi: 10.1371/journal.pone.0337639 (PMC12857961; doi:10.1371/journal.pone.0337639)
Supplement: S1 File — (PDF) [file pone.0337639.s003.pdf]

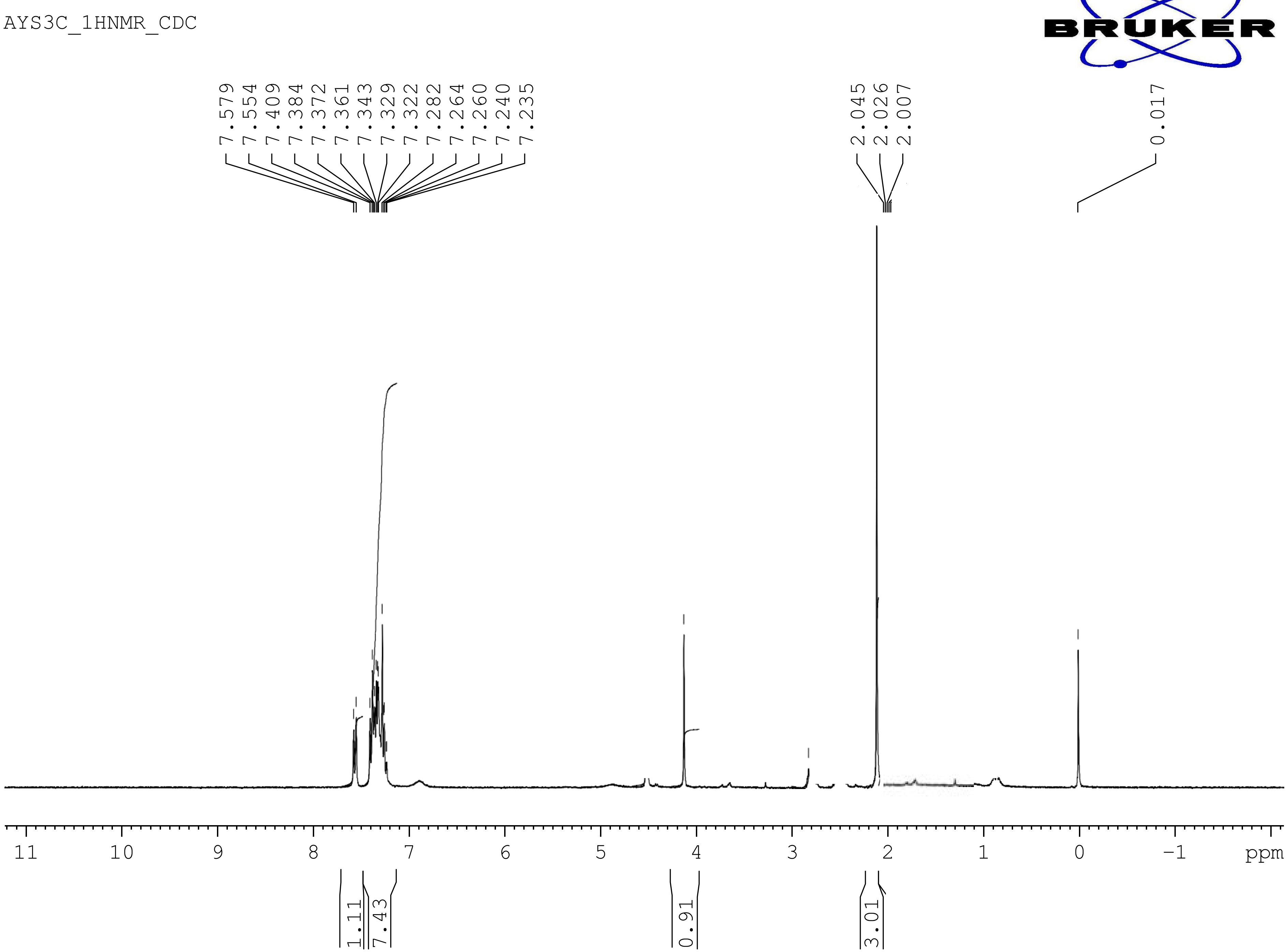
AYS3C_1HNMR_CDC
7.579
7.554
7.409
7.384
7.372
7.361
7.343
7.329
7.322
7.282
7.264
7.260
7.240
7.235
2.045
2.026
2.007
0.017
11
10
9
8
7
6
5
4
3
2
1
0
-1
ppm
1.11
7.43
0.91
3.01

AYS3C_1HNMR_CDC

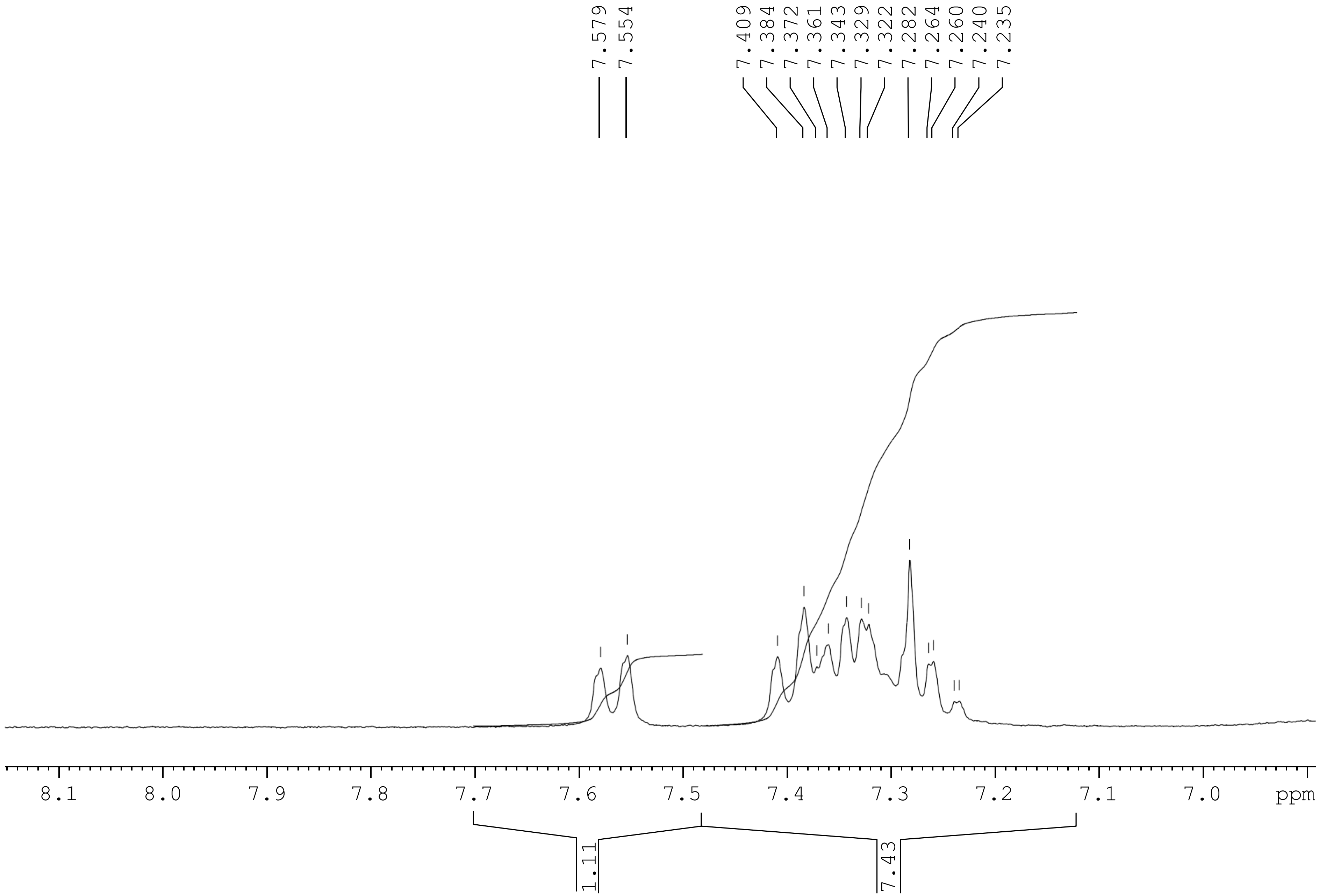

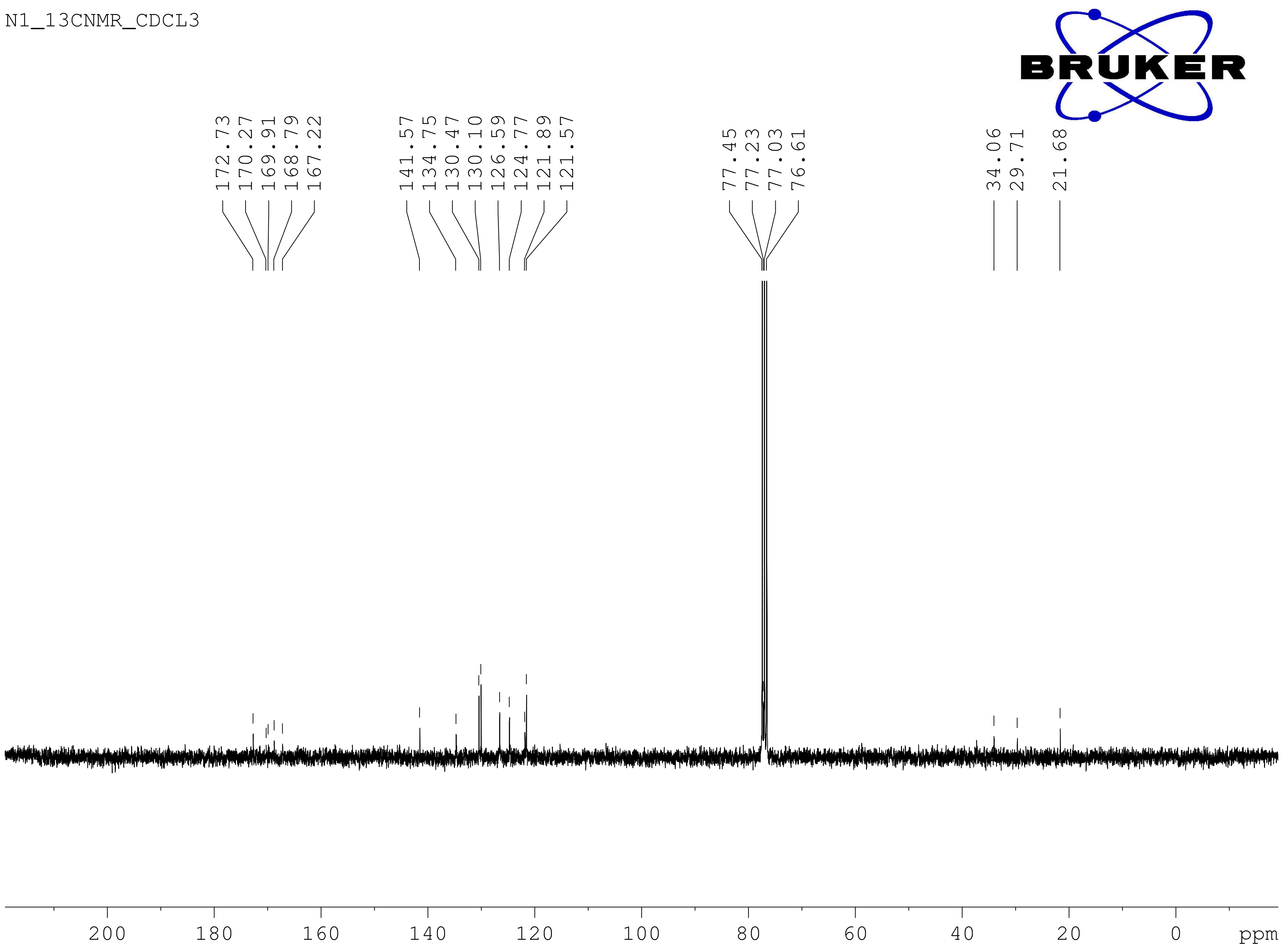
N1_13CNMR_CDCL3
172.73
170.27
169.91
168.79
167.22
141.57
134.75
130.47
130.10
126.59
124.77
121.89
121.57
77.45
77.23
77.03
76.61
34.06
29.71
21.68
200
180
160
140
120
100
80
60
40
20
0
ppm

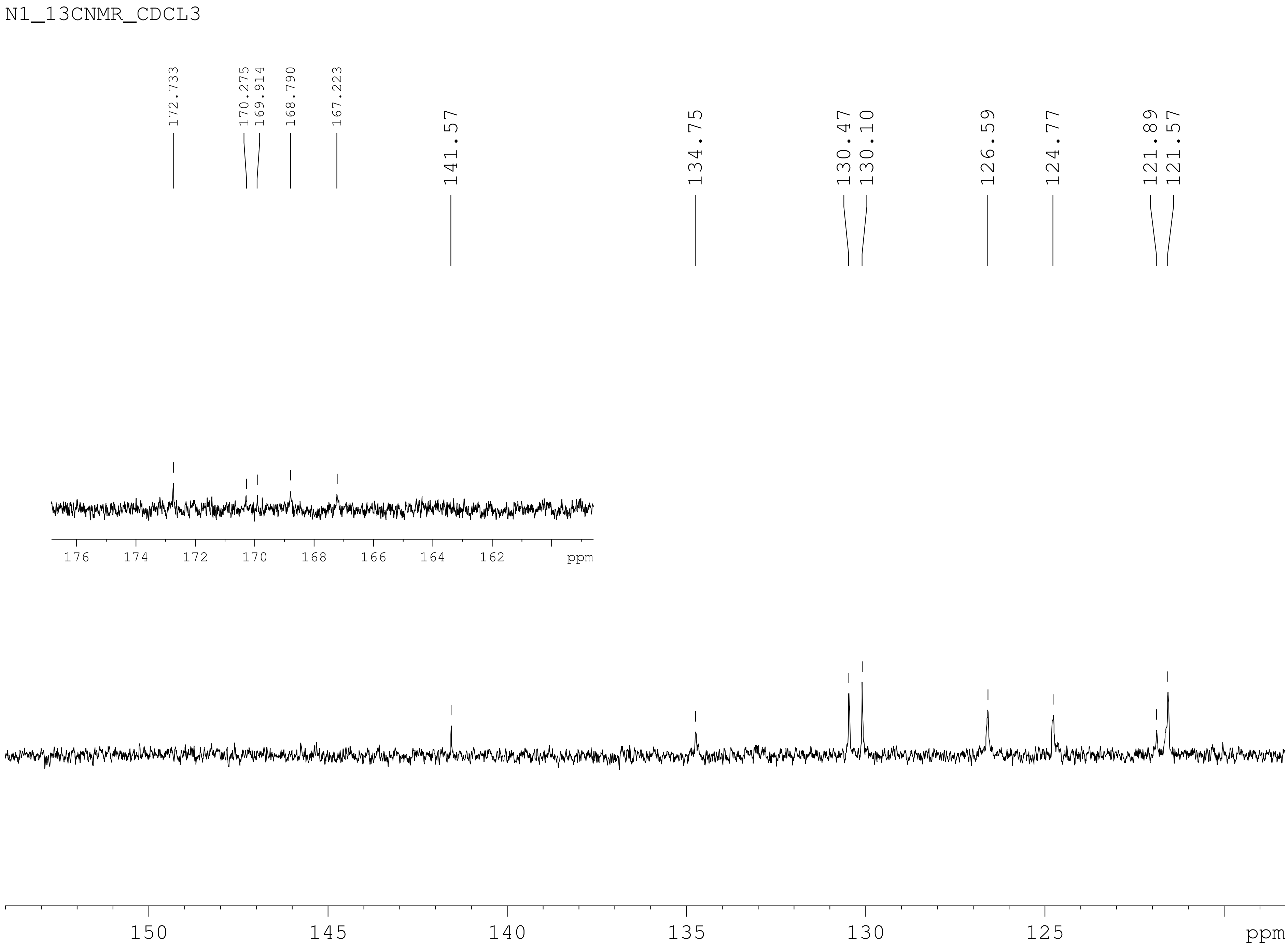
N1_13CNMR_CDCL3
172.733
170.275
169.914
168.790
167.223
141.57
134.75
130.47
130.10
126.59
124.77
121.89
121.57
176
174
172
170
168
166
164
162
ppm
150
145
140
135
130
125
ppm

AYSC2_1HNMR_CD

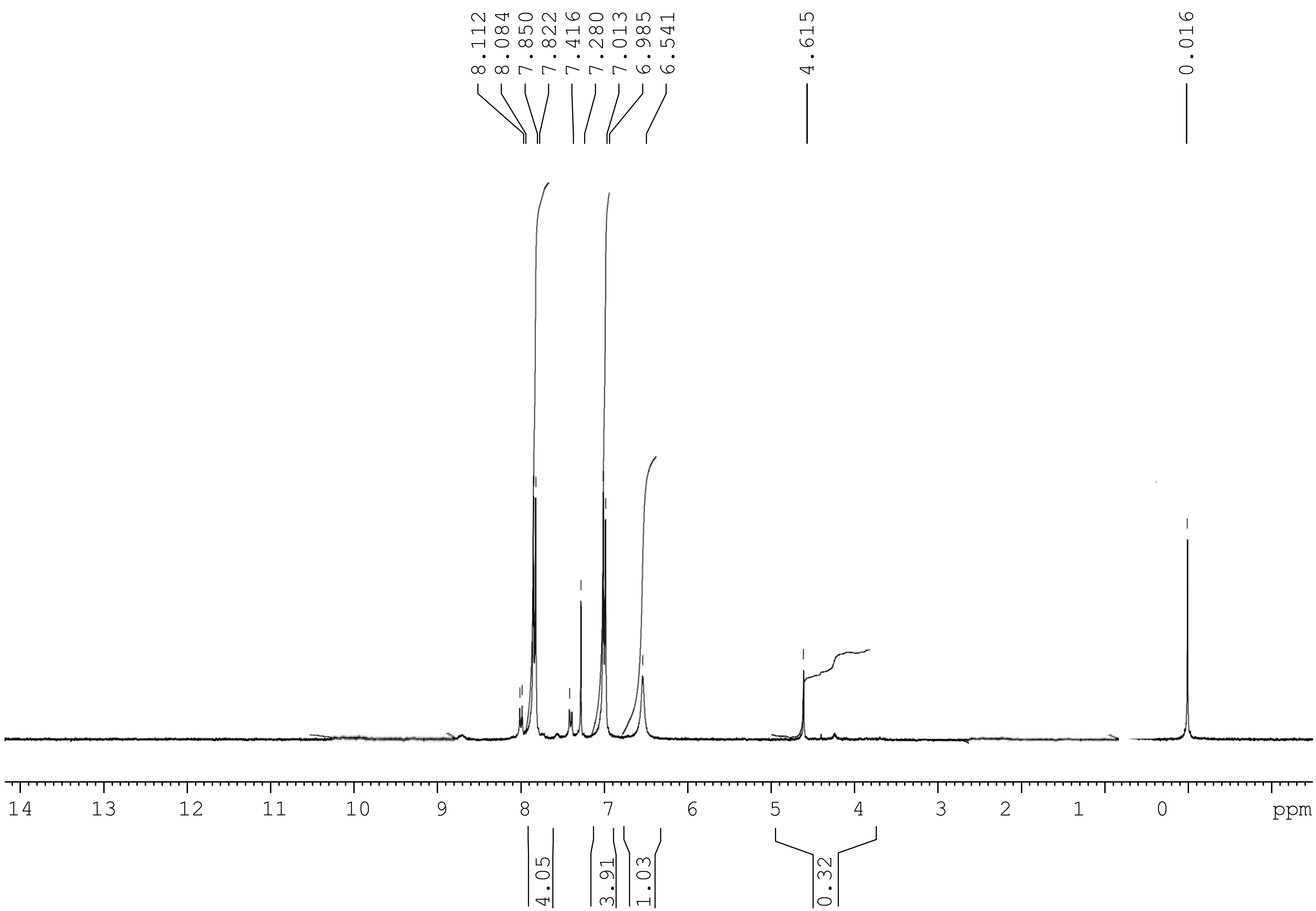

AYSC2_13CNMR_CDCL3

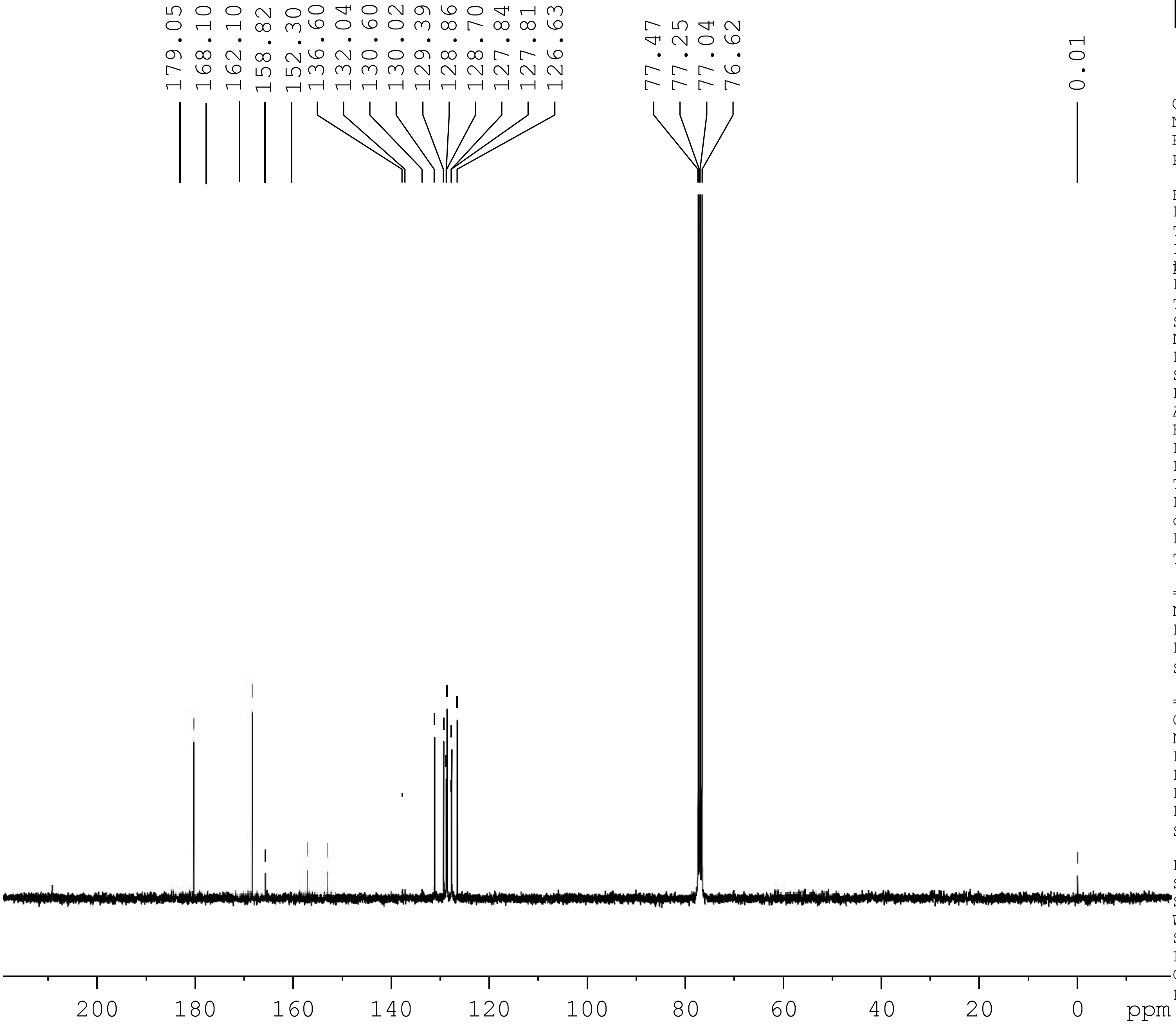

Current Data Parameters
NAME AYSC2_13CNMR_CDCL3
EXPNO 1
PROCNO 1

F2 - Acquisition Parameters
Date_ 20250712
Time 20.20
INSTRUM spect
PROBHD 5 mm BBO BB-1H
PULPROG zgpg30
TD 35968
SOLVENT CDCl3
NS 5120
DS 0
SWH 17985.611 Hz
FIDRES 0.500045 Hz
AQ 0.9999604 sec
RG 4096
DW 27.800 usec
DE 6.00 usec
TE 293.6 K
D1 2.00000000 sec
d11 0.03000000 sec
DELTA 1.89999998 sec
TD0 1

======== CHANNEL f1 ========
NUC1 13C
P1 6.00 usec
PL1 -5.00 dB
SFO1 75.4752953 MHz

======== CHANNEL f2 ========
CPDPRG2 waltz16
NUC2 1H
PCPD2 80.00 usec
PL2 2.00 dB
PL12 20.98 dB
PL13 20.00 dB
SFO2 300.1312005 MHz

F2 - Processing parameters
SI 32768
SF 75.4677490 MHz
WDW EM
SSB 0
LB 1.00 Hz
GB 0
PC 1.40

AYSC3_13CNMR_CDCL3

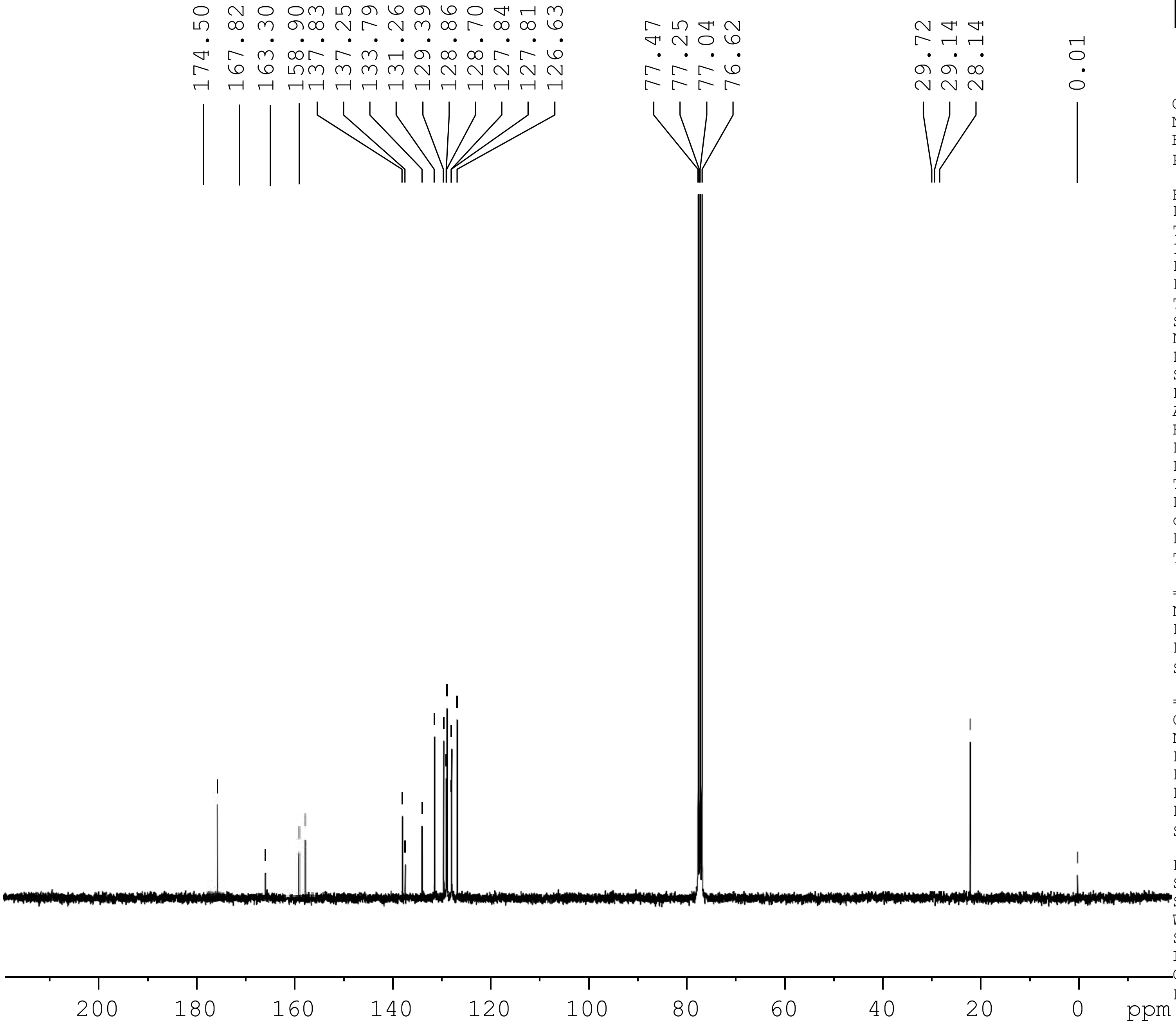

Current Data Parameters
NAME AYSC3_13CNMR_CDCL3
EXPNO 1
PROCNO 1

F2 - Acquisition Parameters
Date_ 20250711
Time 20.35
INSTRUM spect
PROBHD 5 mm BBO BB-1H
PULPROG zgpg30
TD 35968
SOLVENT CDCl3
NS 5120
DS 0
SWH 17985.611 Hz
FIDRES 0.500045 Hz
AQ 0.9999604 sec
RG 4096
DW 27.800 usec
DE 6.00 usec
TE 293.6 K
D1 2.00000000 sec
d11 0.03000000 sec
DELTA 1.89999998 sec
TD0 1

======== CHANNEL f1 ========
NUC1 13C
P1 6.00 usec
PL1 -5.00 dB
SFO1 75.4752953 MHz

======== CHANNEL f2 ========
CPDPRG2 waltz16
NUC2 1H
PCPD2 80.00 usec
PL2 2.00 dB
PL12 20.98 dB
PL13 20.00 dB
SFO2 300.1312005 MHz

F2 - Processing parameters
SI 32768
SF 75.4677490 MHz
WDW EM
SSB 0
LB 1.00 Hz
GB 0
PC 1.40

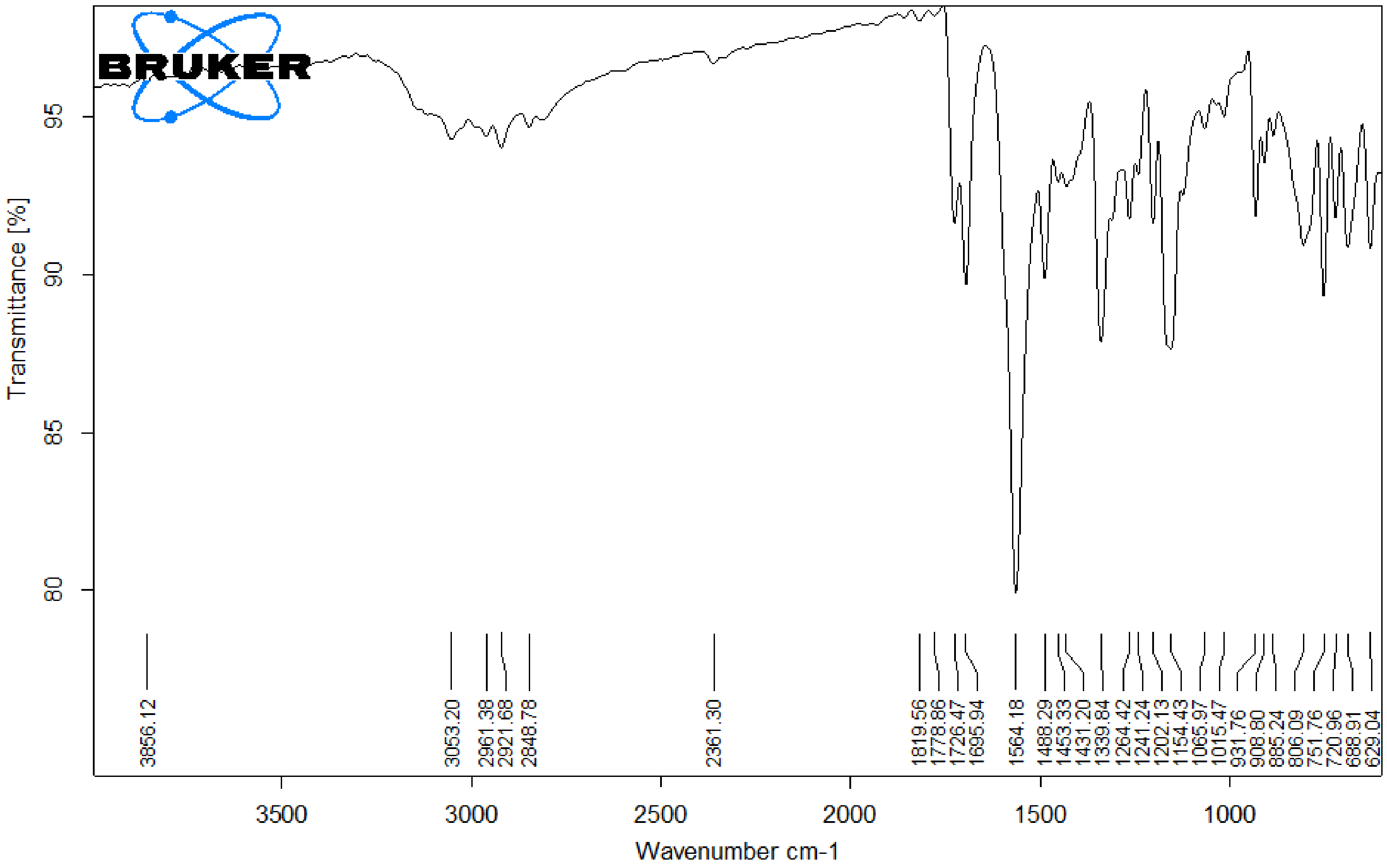

C:\Users\Riphah-Lab\Documents\Bruker\OPUS_7.5.18\DATA\MEAS\Aisha (k-3).0	Aisha (k-3)	Instrument type and / or accessory	10/10/2021

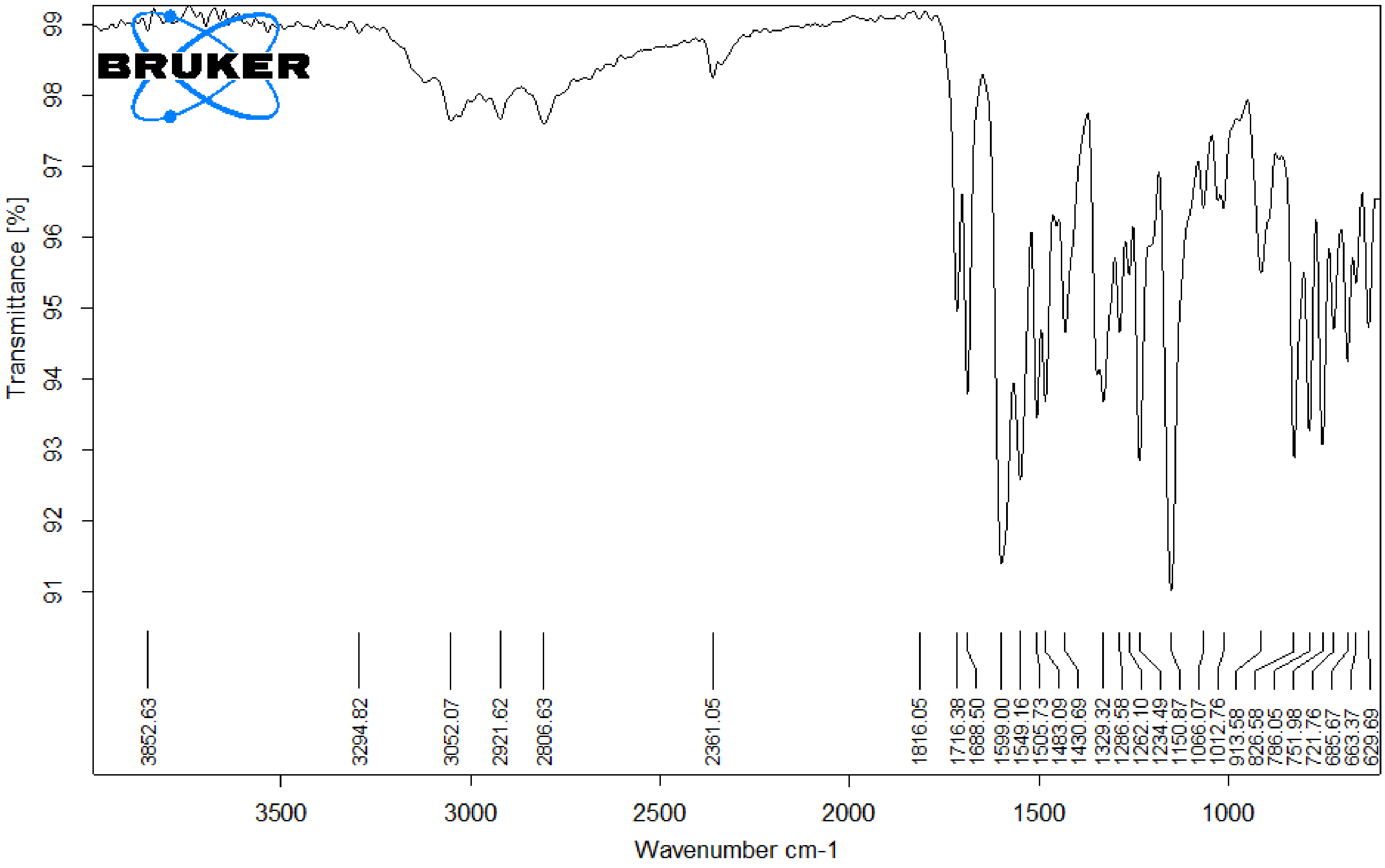

C:\Users\Riphah-Lab\Documents\Bruker\OPUS_7.5.18\DATA\MEAS\Aisha (a-4).0	Aisha (a-4)	Instrument type and / or accessory	10/10/2021
